# An individualized patient-reported outcome measure (PROM) based patient decision aid and surgeon report for patients considering total knee arthroplasty: protocol for a pragmatic randomized controlled trial

**DOI:** 10.1186/s12891-019-2434-2

**Published:** 2019-02-23

**Authors:** Nick Bansback, Logan Trenaman, Karen V. MacDonald, Gillian Hawker, Jeffrey A. Johnson, Dawn Stacey, Deborah A. Marshall

**Affiliations:** 1 School of Population and Public Health, University of British Columbia, Vancouver, BC Canada; 2 Centre for Health Evaluation and Outcome Sciences, Vancouver, BC Canada; 3 Arthritis Research Canada, Richmond, BC Canada; 4 Department of Community Health Sciences, University of Calgary, Calgary, AB Canada; 5 Department of Medicine, University of Toronto, Toronto, ON Canada; 6 School of Public Health, University of Alberta, Edmonton, AB Canada; 7 School of Nursing, University of Ottawa, Ottawa, ON Canada; 8 Centre for Practice Changing Research, Ottawa Hospital Research Institute, Ottawa, ON Canada

**Keywords:** Osteoarthritis, Total knee arthroplasty, Patient-reported outcome measures, Decision quality

## Abstract

**Background:**

While the rates of total knee arthroplasty (TKA) continue to rise worldwide, there are concerns about whether all surgeries are appropriate. Guidelines for appropriateness suggest that patients should have realistic expectations for total knee arthroplasty (TKA), and that the patient and their surgeon should agree that the potential benefits outweigh the potential harms. The objective of this study is to evaluate whether routinely collected pre- and post-TKA patient-reported outcome measures (PROMs) could be integrated into a patient decision aid to better inform these appropriateness criteria. This randomised trial will evaluate the preliminary efficacy of a tailored PROM-based patient decision aid and surgeon report (compared to usual care) for patients considering TKA on decision quality.

**Methods:**

This is a pragmatic, randomised controlled trial conducted at one site in Alberta, Canada. Adults over the age of 30 years, who have been scheduled for a TKA consultation at the Edmonton Bone and Joint Centre with a participating surgeon, who understand, speak, and read English, and can provide informed consent, are eligible to participate. Participants will be randomised to receive a PROM-based patient decision aid and surgeon report before their surgical consultation or usual care. The decision aid will provide patients with information on their expected outcomes based on the EQ-5D-5L PROM, and these estimates are individualized based on clinical and demographic characteristics. The primary outcome of this trial is decision quality. Analysis will consider outcomes intention to treat, and feasibility outcomes for implementing the trial to routine practise.

**Discussion:**

This patient decision aid and surgeon report intervention could contribute to improved treatment decision-making for patients considering total knee arthroplasty.

**Trial registration (registry and number):**

ClinicalTrials.gov: NCT03240913. Registered on August 1, 2017.

## Background

Rates of total knee arthroplasty (TKA) continue to rise worldwide. [1, 2] While TKA reduces pain and disability in a majority of patients, [3] there are concerns about whether all surgeries are appropriate. [3] Six criteria have been endorsed to assess TKA appropriateness. [4] While the surgeon is the best judge for several of these criteria (i.e., the patient has evidence of arthritis on examination; the patient has had an adequate trial of nonsurgical arthritis treatment; the patient is physically and mentally ready to have surgery), two require the elicitation of the patient’s knowledge and preferences. For example, one criterion requires the patient to have “expectations for joint replacement surgery [that] are achievable.” In general, evidence suggests that patients tend to overestimate the potential for benefit of surgery and underestimate the potential for complications. [5] While pre-operative expectations are the strongest predictor of satisfaction with total joint replacement, [6, 7] with as many as one in five patients reporting dissatisfaction with surgery, [7, 8] it can be challenging to elicit patients' expectations. Another appropriateness criterion stipulates that “…the patient and surgeon agree that the potential benefits to the patient of joint replacement surgery outweigh the potential surgical risks.” In order to meet this criterion, patients must be informed about alternatives to TKA and been clear on what matters most to them so they can make informed trade-offs on the relative benefits and risks. Evidence suggests that alternative treatment options, including exercise, education, dietary advice, use of insoles, and pain medication, are not always offered and thus underutilized. [9, 10]

Patient decision aids are interventions that can inform appropriateness criteria for TKA, potentially improving the quality of decisions about appropriate use by supporting shared decision-making between patients and their providers. [11, 12] Patient decision aids in total joint arthroplasty have been found to: increase patient knowledge, result in more realistic expectations, [12, 13] improve patient/provider communication, [14] result in fewer patients having surgery, [12, 13, 15] and be a cost-effective use of resources. [16] While the role of patient decision aids in TKA is promising, there are limitations. Notably, previously developed decision aids are not individualised for each patient, instead relying on average risk information. In addition, they tend to provide information on a narrow set of clinically focussed outcomes (e.g., operative mortality) but not on how treatment will influence outcomes such as pain, which is a clear priority for patients, [17] in addition to mobility, ability to self-care, and ability to participate in activities of daily living [18].

This study seeks to investigate whether the self-reported outcomes of patients who previously underwent TKA can be used to improve decision quality about appropriate use of TKA. Many health systems have been routinely-collecting patient-reported outcome measures (PROMs) pre and post TKA [18–20]. While these data have been collected to support decision-making at a *health systems level,* we believe there is a role for these data to inform setting realistic expectations for patients, and promoting shared decision-making with their care provider. Our preliminary focus group work found that information on ‘average’ patients provided in a decision aid lacked both salience and validity as individuals may perceive that they are different than the average patient (e.g., younger, healthier), and their expectations may truly differ from the average. [21] Moreover, patients were interested in how treatment could impact their day-to-day life - outcomes typically not included in decision aids. [21] Databases of routinely collected PROMs often include more patients than comparable randomised controlled trials, which, when combined with information on demographic and clinical characteristics, may enable outcome estimates to be tailored to the individual. [22] Furthermore, PROMs take the perspective of the patient, and typically represent outcomes that matter more to patients, such as impact on usual activities and self-care, and are described in a language that is easier to understand than clinical measures.

## Methods

The SPIRIT (Standard Protocol Items for Randomized Trials) recommendations guided the development of this protocol. [23] The decision aid was developed according to International Patient Decision Aid Standards (IPDAS). [24]

### Study setting and eligibility criteria

**Fig. 1:**
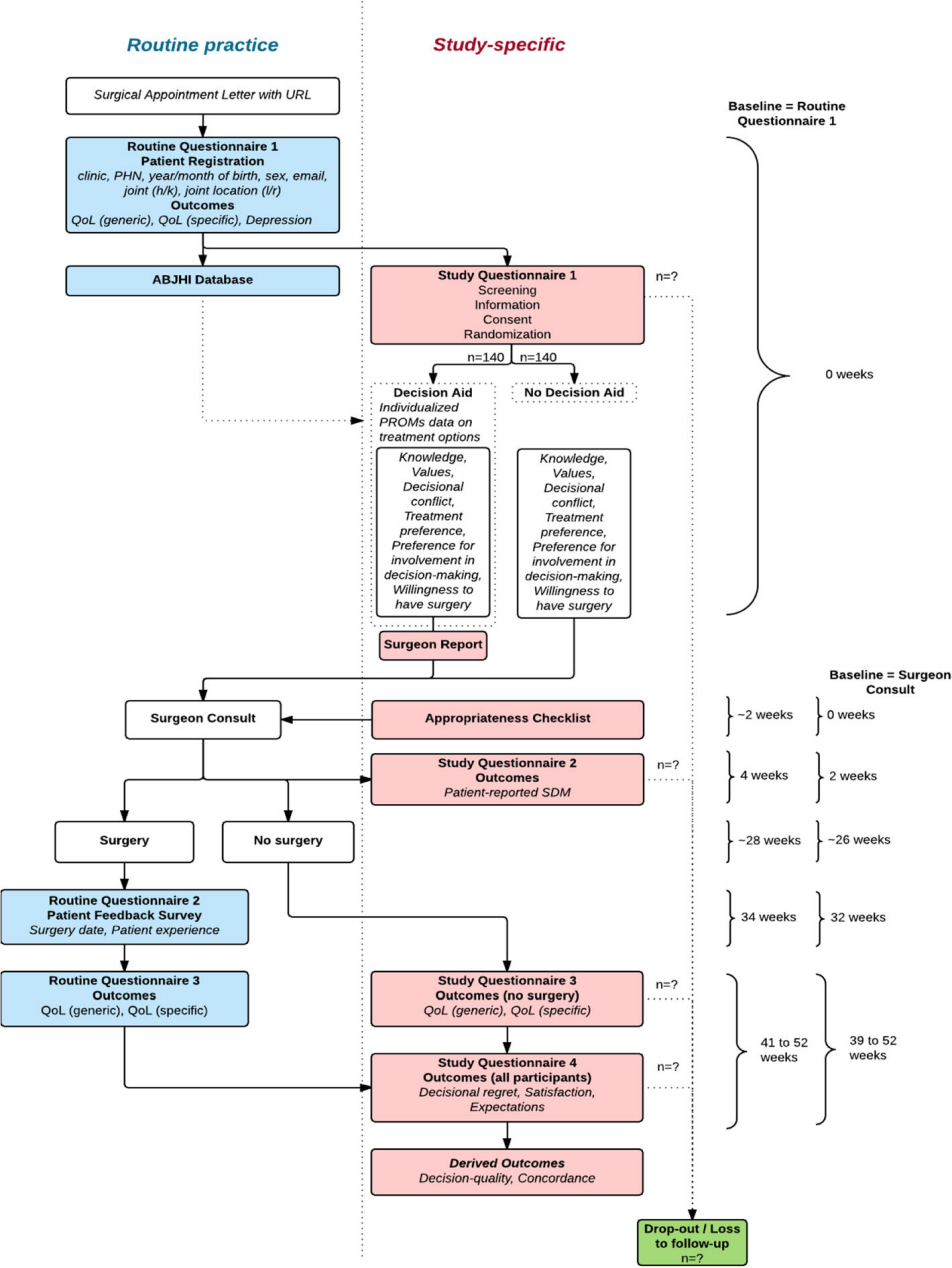
Routine practice and study-specific procedures. PHN: Personal Health Number; h: hip; k: knee; l: left; r: right; QoL: Quality of Life; ABJHI: Alberta Bone and Joint Health Institute; SDM: Shared Decision Making

This study is a prospective, single site, two-arm, pragmatic, randomized controlled trial (RCT). It is based at the Edmonton Bone and Joint Centre in Alberta, Canada. In routine practice, patients are referred to the Edmonton Bone and Joint Centre from their primary care provider, or in a minority of cases from a physiotherapist or surgeon. A proportion of patients undergo screening, to determine whether they are eligible for surgery. Candidates for surgery are scheduled for a surgical consultation with an orthopedic surgeon, with most patients waiting 6 to 8 weeks. Most patients receive an appointment reminder letter 3–4 weeks prior to this consultation, which also asks them to complete an online questionnaire at a specified URL (*Routine Questionnaire 1*, Fig. 1). Approximately 30% of participants complete this questionnaire prior to arriving for their consultation, with the remainder completing it while in the waiting room. Following their surgical consultation, patients may be scheduled for surgery (average wait time of 5 to 6 months), or choose not to go on the waiting list. For those that undergo surgery, there is additional data collected on their surgical experience at six-weeks post-surgery (*Routine Questionnaire 2*, Fig. 1) and their outcomes at 3-months post-surgery (*Routine Questionnaire 3*, Fig. 1) and 1-year post-surgery. Currently, there is no follow-up of patients who choose not to have surgery. This study will be integrated into the clinical workflow at the Edmonton Bone and Joint Centre. It aims to evaluate the influence of a PROM-based patient decision aid and surgeon report in patients considering TKA (*decision aid and surgeon report arm*), compared to a control group, which follows routine practice (*no decision aid arm*), on decision quality. Participants will be recruited to the trial following completion of *Routine Questionnaire 1* (Fig. 1), which includes basic demographic information, and information about the affected joint(s). Participants will be excluded if they have not completed the *Routine Questionnaire 1* online prior to the surgical consultation. Patients will be linked to *Study Questionnaire 1* (Fig. 1), which will screen participants for their eligibility. Eligible study participants include: adults over the age of 30 years, who have been scheduled for a TKA consultation at the Edmonton Bone and Joint Centre with a participating surgeon (18 out of 23 surgeons at the clinic), understand, speak, and read English, and can provide informed consent. Patients are excluded if they have had a prior TKA, or have physician-diagnosed rheumatoid arthritis, psoriatic arthritis, ankylosing spondylitis, fibromyalgia or gout. Eligible participants will be informed about the trial via the study-specific web URL that is provided on the appointment reminder letter, and asked to provide online consent. Those consenting to participate will be randomised to either the *decision aid and surgeon report arm* or the *no decision aid arm*. Participants not eligible for inclusion and those who decline consent will follow routine practice.

### Decision aid and surgeon report arm (Fig. 1)

**Fig. 2:**
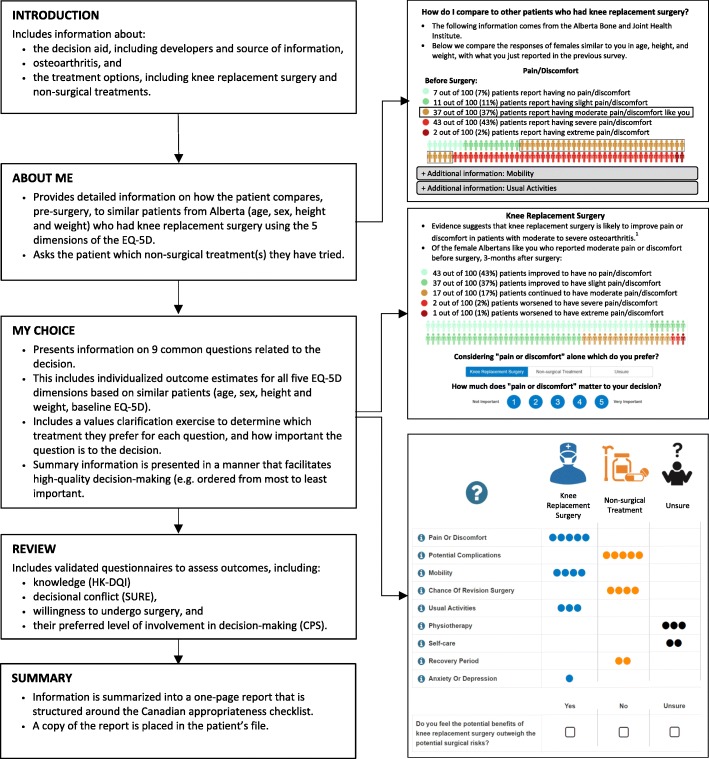
Overview of PROM-based decision aid. EQ-5D: EuroQol Questionnaire 5-Dimensions; HK-DQI: Hip and Knee Decision Quality Instrument; CPS: Control Preferences Scale

The decision aid and surgeon report arm consists of an online, tailored, PROM-based patient decision aid for the patient and a summary report for the surgeon (Fig. 2). The decision aid has been developed following IPDAS criteria. [24] It is built on existing software which allows information to be tailored to users, and described in a way that promotes higher-quality decision-making. [25] The decision aid includes information about osteoarthritis (OA), which is the primary indication for over 95% of TKAs, and the treatment options available: knee replacement surgery, or other guideline recommended non-surgical treatment (e.g., weight loss, physical therapy, prescription pain medication). [26] These two treatment options are compared across nine aspects that previous qualitative studies have determined to be key factors influencing decision-making. These include the five dimensions from the routinely used EQ-5D PROM related to health-related quality of life (HRQoL): *mobility, self-care, usual activities, pain/discomfort,* and *anxiety/depression,* [27] in addition to: chance of repeat surgery, physiotherapy, recovery period, and risks. Evidence for each aspect comes from both the literature, and data routinely collected in Alberta. Patients are presented with information on: how they compare (pre-surgery) on these five HRQoL dimensions relative to similar patients (based on age, sex, body mass index), and the 3-month post-surgical outcomes of similar patients (based on the above characteristics and baseline HRQoL).

To date, the Alberta database includes HRQoL data over 6000 individuals who have undergone TKA. The pre-surgery matching algorithm began by searching for more than 50 patients of the same sex, within 5 years of age, and 2.5 body mass index points. These conditions were relaxed in a stepwise fashion until more than 50 matches were found. The pre-surgery information from the matches was presented in 5 separate figures, which allowed patients to compare themselves against similar patients (i.e., am I experiencing more, less, or about the same amount of pain or discomfort as similar patients who undergo surgery?). The post-surgery matching algorithm was completed using the same conditions, in addition to matching patients on self-reported baseline HRQoL. Matching was completed independently for each HRQoL dimension (i.e., there were different number of matches for different dimensions), and results were presented in 5 figures. Figures displayed the outcomes of similar patients 3-months following TKA. For example, patients with ‘moderate pain or discomfort’ at baseline would see the proportion of similar patients who: improved to have no or slight pain or discomfort, continued to have moderate pain or discomfort, or worsened to have severe or extreme pain or discomfort.

The information collected from each participant in the decision aid will automatically generate a summary report for the surgeon, which will be printed by a research assistant in the clinic, and placed in the participant’s file along with an appropriateness checklist. [4] The summary report will be available for viewing by the surgeon during the consultation and the appropriateness checklist will be completed by the surgeon during or after the consultation.

### No decision aid arm (Fig. 1)

The no decision aid arm will follow routine practice. This includes routine data collection, including several PROMs, but no information on OA, treatment options, or potential outcomes. The only deviations from routine practice include the collection of study-specific questions for informing the effectiveness endpoints and the provision of a printed appropriateness checklist in the patients file by a research assistant in the clinic for completion by the surgeon during the surgical consultation.

### Outcomes

#### Primary outcome

The primary outcome for this trial is the difference in the proportion of participants making quality decisions between the two arms. Decision quality is determined to be the key outcome for establishing the effectiveness of decision-making interventions, [11] and is defined as “the extent to which patients are informed and receive treatments that reflect their goals and treatment preferences.” [28] A quality decision requires the patient to: a) be knowledgeable, and b) make a decision that is in concordance with their values. [28] Concordance requires that the patients’ values match with the decision that was implemented, defined as whether the patient had surgery or not at the final assessment. To measure knowledge, we modified one question from the 5-item Hip and Knee Decision Quality Instrument (HK-DQI) related to pain when walking after surgery. In the HK-DQI this question reads, “If 100 people have hip/knee replacement surgery, about how many will have less hip/knee pain when walking after the surgery?” Given the overlap with the EQ-5D-5L dimension of pain or discomfort, and that the decision aid provides tailored estimates of outcomes based on clinical and demographic factors, this question was modified to, “If 100 people like you have knee replacement surgery, how many people improve and have NO or SLIGHT pain or discomfort after surgery?” Individuals getting three or more correct responses (out of five) are considered knowledgeable. Concordance will require that the patient’s treatment preference match their chosen treatment. The following question will measure treatment preference: “Do you feel the potential benefits of knee replacement surgery outweigh the potential surgical risks?” Response options include: “yes,” “no,” and “unsure.” Treatment choice will be ascertained through chart review.

#### Secondary outcomes

Secondary outcomes are outlined in Table 1 and include those that evaluate preliminary effectiveness of the decision aid, and those related to feasibility of the trial.

**Table 1:** Outline of outcomes, instruments, and data collection

	Instrument Name	Source	Baseline (t_0_)	Post-consult (t_1_)	Follow-up (t_2_)
ENROLMENT					
Eligibility screen			X		
Informed consent			X		
Randomization			X		
INTERVENTIONS					
Decision aid plus surgeon report arm (decision aid, surgeon report, appropriateness checklist)			X		
No decision aid arm (appropriateness checklist)			X		
ASSESSMENTS					
Decision quality * Primary Outcome	Modified version of the HK-DQI	[28]			DQ_2_ [K_0_, Pref_0_, Surg_2_]
Demographics (Birthdate, Sex, Joint)			D_0_		
QoL (Generic)	EQ-5D-5L	[29]	E_0_		E_2_
QoL (Condition-specific)	WOMAC	[31]	W_0_		W_2_
Depression	PHQ-9		P_0_		
Knowledge	Modified version of the HK-DQI	[28]	K_0_		
Decisional conflict	SURE	[32]	DC_0_		
Treatment preference		[4]	Pref_0_		
Preference for involvement in decision-making	CPS	[33]	CPS_0_		
Willingness to have surgery			Will_1_		
Patient-reported shared decision-making	CollaboRATE	[34, 35]		C_1_	
Decisional regret	Decision Regret Scale	[36]			R_2_
Satisfaction		[7]			S_2_
Expectations		[7]			EXP_2_
Surgical consult	EMR			Consult_1_	
Surgery	EMR				Surg_2_
Concordance	HK-DQI	[28]			Con_2_ [Pref_0_, Surg_2_]
Feasibility: Recruitment			Recruit_0_		
Feasibility: Questionnaire Completion			Complete_0_	Complete_1_	Complete_2_

QoL: Quality-of-Life; EQ-5D-5L: EuroQol 5-Dimensions Questionnaire (5-level); PHQ-9: Patient Health Questionnaire; WOMAC: Western Ontario and McMaster Universities Arthritis Index; HK-DQI: Hip and Knee Decision Quality Instrument; CPS: Control Preferences Scale; EMR: Electronic Medical Record

HRQoL will be assessed by the EQ-5D-5L instrument, [27] which is a generic instrument that has been demonstrated to be valid and reliable in arthritis patients, [29] and the condition-specific Western Ontario and McMaster Osteoarthritis Index (WOMAC) instrument. [30] The WOMAC is widely used to evaluate changes in quality-of-life for people with osteoarthritis, and specifically for those undergoing TKA. It includes three subdomains related to pain, stiffness, and physical functioning, and has been shown to be reliable and valid in a variety of patient groups and across a range of interventions. [31] Depression at baseline will be assessed by the Patient Health Questionnaire (PHQ-9). Treatment preference will be elicited based on a question from the appropriateness checklist which asks whether the patient feels the potential benefits of surgery outweigh the potential surgical harms. [4] We will also include the SURE measure of decisional conflict, which includes four additional items that measure personal perceptions of uncertainty in choosing options, factors contributing to uncertainty, and effective decision-making. SURE been shown to be both internally consistent and reliable (test-retest exceeded 0.78), is correlated with the constructs of knowledge, regret and discontinuance, and has the ability to discriminate between those who make and delay decisions. [32] Willingness to have surgery will be assessed using a single item question.

Preference for involvement in decision-making will be assessed by the Control Preferences Scale (CPS) which ranges from “I prefer to make the final treatment decision” to “I prefer to leave all treatment decisions to my doctor.” [33] Patient-reported shared decision-making will be assessed using the CollaboRATE scale, a validated patient-reported measure which measures shared decision making through three brief questions. [33, 34] Decisional regret will be assessed by the 5-item decisional regret scale, [36] and patient satisfaction will be assessed using a validated three-item instrument. [37] In patients who had surgery, we will assess whether the expectations of surgery were met with a question that has been used previously in joint replacement. [7] Lastly, feasibility will be assessed based on recruitment rates, and rate of questionnaire completion pre and post surgery.

### Participant timeline (Fig. 1)

There are three assessments in this trial: baseline (pre-surgical consultation), post-surgical consultation, and follow-up. Since this RCT has been designed to fit within routine practice at the Edmonton Bone and Joint Centre, study specific outcomes will be collected and combined with routine data collection. This will ensure that no data collection is done in duplicate, thereby easing the measurement burden. It will also ensure that the patient decision aid could be integrated into routine practice if proven effective.

#### Baseline assessment (t0)

After completing *Routine Questionnaire 1* (Fig. 1), participants will be screened to determine if they have been scheduled to visit a participating surgeon, and for eligibility in the trial. Eligible participants will be asked for consent to participate in the study, and complete *Study Questionnaire 1* (Fig. 1), either in the decision aid and surgeon report arm or the no decision aid arm. The timing of this assessment will be dependent on when the patient decides to complete the online questionnaire. When the patient attends their surgical consultation, a research assistant will attach a copy of the summary report and appropriateness checklist (decision aid and surgeon report arm) or appropriateness checklist (no decision aid arm) to the patient’s file.

#### Post-consultation assessment (t1)

Following the surgical consultation (approximately 2-weeks post-consult), all participants will be sent an e-mail asking them to complete *Study Questionnaire 2* (Fig. 1) at their convenience. This assessment will ask questions about the surgical consultation and the participant’s involvement in decision-making.

#### Follow-up assessment (t2)

The final follow-up point (3-months post-surgery) will assess a variety of outcomes. For those who had surgery, they will be asked to complete *Routine Questionnaire 3*, while those that did not have surgery will complete *Study Questionnaire 3* at approximately 1 year from baseline (Fig. 1). Both these surveys will assess the patients’ health status (EQ-5D and WOMAC). All participants will then complete *Study Questionnaire 4* (Fig. 1), which assesses patient satisfaction, and some study-specific outcomes including: decisional regret and whether expectations were met. All data collection will be completed through online questionnaires.

### Sample size and recruitment

A previous decision aid trial in hip and knee arthroplasty found that decision quality improved from 44.5 to 56.1% through the use of a patient decision aid and surgeon report. [12] We expect that the current tool will have a greater impact on decision quality through the inclusion of tailored outcome information for each participant based on data from Alberta collected over the past 3 years, rather than presenting average outcome information for all patients. A sample size of 127 participants per arm are needed, to detect an absolute improvement in decision quality of 17.5% (an increase in decision quality from 44.5 to 62%), using a two-sided t-test at the 5% level of significance with 80% power. To account for a 10% loss to follow-up, our target enrolment is 140 patients per arm for a total of sample size of 280. Participants will be recruited from the Edmonton Bone and Joint Centre until the target sample of 280 is achieved. Based on a previous trial run at this centre, [38, 39] we will aim to recruit 40 individuals per month, requiring approximately 7 months to achieve the desired sample size.

### Assignment of interventions

Participants will be randomly assigned to either the decision aid or no decision aid arm in a 1:1 allocation using a computer-generated randomization schedule with permuted blocks of random sizes. The randomization will be conducted in Research Electronic Data Capture (REDCap) software. All participants who meet the inclusion criteria and consent to participate will be randomized.

### Blinding (masking)

It is not possible to blind the surgeons because patients randomized to the decision aid arm will have a summary report in their surgical file (decision aid arm) or not (no decision aid arm). Analysts will be blinded to treatment allocation until after all participants have had their surgeon visits. This will enable some secondary analyses to be performed. At this time point, analysts will not have access to follow-up data.

### Data collection, management, and analysis

#### Data collection methods and data management

Most data collection will be completed electronically and stored on secure servers at the Alberta Bone and Joint Health Institute (ABJHI). Data entry will be facilitated through two platforms: REDCap, and the PROM-based decision aid software. Data will be entered electronically by patients and the appropriateness questionnaire will be completed by surgeons on paper and then entered electronically into the REDCap database by a research assistant. The anonymized data will be stored in a database on a web server that is only accessible to the necessary ABJHI staff and the research project team.

ABJHI has robust controls in place to ensure the security and privacy of identifiable healthcare information; these include physical controls and technological controls. Their security protocols are compliant with requirements set by the University of Calgary, Alberta Health Services, and the Health Insurance Portability and Accountability Act. All electronic identifiable information is secured on ABJHI servers in either folders with permission restricted to the network administrator, or in a relational database with access restricted to the database administrator. Wireless network access is encrypted using secure enterprise WPA2 encryption.

### Statistical methods (Table 2)

The primary analysis evaluating decision quality will be by intention-to-treat. There is no risk of participants in the no decision aid arm receiving the decision aid intervention. However, some participants randomized to the patient decision aid may not complete or engage with the tool in a meaningful way. This includes participants who click through the decision aid without spending sufficient time to read the material. A secondary analysis will assess decision quality while excluding those that do not engage, determined by a minimum of 20 min to complete the decision aid. We will analyze the participants who are lost-to-follow-up using multiple imputation to fully understand the uncertainty due to missing data. Secondary outcomes will be compared as described in Table 2.

**Table 2:** Analysis of primary outcome and secondary outcomes

Outcome	Categorical (Cat) Continuous (Con)	Decision aid arm (DA)	No decision aid arm (RP)	Notes
Decision quality	Cat	DQ_2,DA_	DQ_2,RP_	
Quality of Life (Generic)	Con	E_2,DA_ – E_0,DA_	E_2,RP_ – E_0,RP_	
Quality of Life (Condition-specific)	Con	W_2,DA_ – W_0,DA_	W_2,RP_ – W_0,RP_	
Depression	Con	P_2,DA_ – P_0,DA_	P_2,RP_ – P_0,RP_	
Knowledge	Cat	K_0,DA_	K_0,RP_	Modified version of the 5-item HK-DQI
Decisional conflict	Cat	DC_0i,DA_	DC_0i,RP_	Evaluated for each question individually (i = 1–4)
Treatment preference	Cat	Pref_0,DA_	Pref_0,RP_	
Preference for involvement in decision-making	Cat	CPS_0,DA_	CPS_0,RP_	
Willingness to have surgery	Cat	Will_1,DA_	Will_1,RP_	
Patient-reported shared decision-making	Cat Con	C_1,DA_	C_1,RP_	Top-score: complete SDM vs. not complete Mean-score: complete SDM (12) to no SDM (0)
Decisional regret	Con	R_2,DA_	R_2,RP_	
Satisfaction	Con	S_2,DA_	S_2,RP_	Only for those who had surgery
Expectations	Cat	EXP_2,DA_	EXP_2,RP_	Only for those who had surgery
Surgical consult	Cat	Consult_1,DA_	Consult_1,RP_	
Surgery	Cat	Surg_2,DA_	Surg_2,RP_	
Concordance	Cat	Con_2,DA_	Con_2,RP_	
Feasibility: Recruitment	Con	Recruit_0,DA + RP_		Evaluated for the trial and reported as a percentage
Feasibility: Questionnaire Completion	Con	Complete_0,1,2,DA + RP_		Evaluated for the trial and reported as a percentage

HK-DQI: Hip and Knee Decision Quality Instrument; SDM: Shared Decision Making

Categorical outcomes will be compared between groups using Cochran-Mantel-Haenszel chi-squared tests, with the relative difference in proportions between the decision aid and no decision aid arms calculated as relative risks with 95% confidence intervals. If there is imbalance in covariates previously reported to be associated with surgery uptake, such as age or sex, dichotomous outcomes will be assessed using logistic regression and reported as odds ratios with 95% confidence intervals. We will check the usual statistical assumptions and transform continuous data if necessary. We will use SAS software to fit linear models and use generalized linear models where the assumptions and data distributions are not normally distributed.

Analysis of continuous outcomes will be completed with a 2-sample 2-tailed student’s t-test. As with categorical outcomes, if there is imbalance in key covariates, continuous outcomes will be assessed using linear regression, and if the assumptions do not hold, we will use generalized linear models. Feasibility targets are: a) > 70% recruitment of eligible patients completing their PROMs electronically, and b) > 90% of patients completing study questionnaires.

### Ethics and dissemination

This study has been approved by the University of Calgary and University of British Columbia research ethics boards. Any modifications to this protocol which impact study procedures or analysis will result in an amendment to the protocol and ethics application. There are no plans for ancillary studies. There are no restrictions on dissemination of the results, and we plan to disseminate the results through peer-reviewed publications and at academic conferences.

## Discussion

This study seeks to improve decision quality for appropriate use of TKA by supporting shared decision-making between patients and their provider. This is not the first study of a patient decision aid in TKA. However, it is the first to our knowledge that is using routinely collected patient-reported outcome data to individualize outcomes to participants within routine practise. We hypothesize that individualized outcome data will be more salient than average outcome data, and thereby demonstrate a greater effect on decision-making outcomes. Moreover, the pragmatic design means that the intervention could be more easily implemented into routine practice to those already completing PROMs electronically.

We developed a new decision aid so that the individualized PROMs could be integrated and language made contextual to the situation in Alberta. There is limited evidence on the usability of existing patient decision aids for TKA, and our heuristic evaluation and usability testing discovered numerous limitations with both the navigation and interpretability of information in existing tools. The primary limitation of our decision aid is the lack of PROMs information for the option of not choosing TKA, as most routine PROMs systems tend to only follow patients who have had TKA surgery. The trial will begin the data collection to overcome this limitation, and we hope this will continue to build up sufficient data in the future.

There are other important limitations to this study. Not all surgeons have agreed to participate in the study, and those that do will be aware of the arm to which participants are randomized because of the surgeon report on the patient chart, potentially biasing the results. The use of a research assistant to print out the decision aid summary report limits the pragmatic nature and external validity of the trial. As does excluding participants who do not complete the questionnaires online prior to the surgical consultation. This may be due to patients' lack of access or comfort in using a computer. The rate of participants completing the routine questionnaires online before their surgical consult is increasing over time, so we believe this will be less of a limitation for the future. Enabling participants to have time to work through the decision aid at their own pace and have time to review and engage with the information was considered necessary. The aim in the future is to integrate the summary report automatically into each patient’s electronic medical record, but we believe there needs to be a transition for surgeons to become familiar with the report. Further, our control arm does not include a patient decision aid without PROMs and based on average values. If the trial meets its primary outcome, we propose a future multi-centre study that compares different versions of decision aids to address these limitations.

In summary, we believe the results of this study could have important implications for the use of PROMs in clinical decision-making. In an era where people increasingly rely on the reports of others to guide which hotel they stay in, which movie they watch, and which product they buy, we believe large scale PROM collection is the natural forum for this information to be utilized in health care.

## Abbreviations

ABJHI: Alberta bone and joint health institute
CPS: Control preferences scale
EMR: Electronic medical record
EQ-5D-5L: EuroQol 5 dimensions 5 level
HK-DQI: Hip and knee decision quality instrument
HRQoL: Health-related quality of life
IPDAS: International patient decision aid standards
OA: Osteoarthritis
PHQ-9: Patient health questionnaire
PROM: Patient-reported outcome measure
RCT: Randomized controlled trial
REDCap: Research electronic data capture
SDM: Shared decision making
SPIRIT: Standard protocol items for randomized trials
TKA: Total knee arthroplasty
WOMAC: Western Ontario and McMaster osteoarthritis index
WPA2: Wi-fi protected access II
